# Who keeps on working? The importance of resilience for labour market participation

**DOI:** 10.1371/journal.pone.0258444

**Published:** 2021-10-13

**Authors:** Espen Berthung, Nils Gutacker, Oddgeir Friborg, Birgit Abelsen, Jan Abel Olsen

**Affiliations:** 1 Department of Community Medicine, Faculty of Health Sciences, UIT The Arctic University of Norway, Tromsø, Norway; 2 Centre of Health Economics, University of York, York, United Kingdom; 3 Department of Psychology, Faculty of Health Sciences, UIT The Arctic University of Norway, Tromsø, Norway; 4 Division of Health Services, Norwegian Institute of Public Health, Oslo, Norway; Norwegian University of Science and Technology, NORWAY

## Abstract

**Background:**

It is widely recognized that individuals’ health and educational attainments, commonly referred to as their human capital, are important determinants for their labour market participation (LMP). What is less recognised is the influence of individuals’ latent resilience traits on their ability to sustain LMP after experiencing an adversity such as a health shock.

**Aim:**

We investigate the extent to which resilience is independently associated with LMP and moderates the effect of health shocks on LMP.

**Method:**

We analysed data from two consecutive waves of a Norwegian prospective cohort study. We followed 3,840 adults who, at baseline, were healthy and worked full time. Binary logistic regression models were applied to explain their employment status eight years later, controlling for age, sex, educational attainment, health status at baseline, as well as the occurrences of three types of health shocks (cardiovascular diseases, cancer, psychological problems). Individuals’ resilience, measured by the Resilience Scale for Adults (RSA), entered as an independent variable and as an interaction with the indicators of health shocks. In separate models, we explore the role of two further indicators of resilience; locus of control, and health optimism.

**Results:**

As expected, health shocks reduce the probability to keep on working full-time. While both the RSA and the two related indicators all suggest that resilience increases the probability to keep on working, we did not find evidence that resilience moderates the association between health shocks and LMP.

**Conclusion:**

Higher levels of resilience is associated with full-time work as individuals age.

## 1. Introduction

Health is a crucial determinant of labour market participation (LMP) [1]. Individuals who experience health adversities are less likely to work [2–4], and, if they do work, they are more likely to work fewer hours [5, 6]. However, the heterogeneity in how people respond to health shocks is noteworthy. Studies report that educational attainment is independently associated with a higher probability of returning to work after suffering health shocks [7]. Thus, analyses of LMP would often start by considering variations in individuals’ health and education, i.e. their human capital.

The knowledge of how personality traits influence LMP is less clear. A relevant factor for improving our understanding of the LMP dynamics may be individuals’ resilience. In psychological capital (PsyCap) theory resilience is defined as ‘*the capacity to rebound or bounce back from adversity*, *conflict*, *failure or even positive events*, *progress and increased responsibility*’ [8]. It is used to explain why people exposed to adversity or serious risks continue to function relatively well and maintain their health and well-being [9, 10]. The literature emphasizes two aspects of resilience: i) recovery, which is how well individuals bounce back and recover from adversity [8], and; ii) sustainability, which is the capacity to continue forward after adverse events [11]. Few studies have examined the role of potential resilience indicators for LMP. However, one study by Schurer [12] investigated how *Locus of Control* (LOC) relates with LMP among men who experienced health shocks. The results showed that men with negative control beliefs were 100% more likely to drop out of the labour market a year after the health shock than those with positive control beliefs.

In PsyCap theory, resilience is considered an important component of psychological capital. It therefore may contribute–beyond human capital, to explain variations in LMP. Economic research has found that PsyCap and resilience are positively associated with work engagement [13], job performance [14] and job satisfaction [15]. Conversely, resilience is negatively associated with voluntary absenteeism [16] and burnout [17, 18]. Moreover, research that used resilience as a moderator has found that resilience mitigates the negative effects of job insecurity, such as emotional exhaustion and counterproductive work behaviour [19]. Hence, resilient individuals would better counteract reductions in their *human capital* as caused by a health shock.

In the current study, we expand prior research by testing the hypothesis that higher personal resilience helps individuals sustain their level of LMP as they age. For this purpose, we employ an abbreviated version of a validated resilience measure, i.e. the Resilience Scale for Adults (RSA). In addition, for improving measurement reliability, we also add two variables that is considered as representatives of resilience, i.e., locus-of-control [12, 20, 21] and optimism [20, 22, 23]. In the current study, the two variables emerge as particularly meaningful, in that they were specifically referring to locus of control *at work*, and optimism with regard to one’s future *health*. Furthermore, we examine the hypothesis that resilience operates as a protective factor, i.e. whether it moderates the presumed negative association between health shock and LMP. We provide new evidence about LMP in an institutional setting characterised by generous welfare arrangements for people who may have limited capacity to work.

## 2. Method

### 2.1 Material

We used data from the Tromsø Study, which is a prospective cohort study of the adult population residing in the municipality of Tromsø. With around 78,000 inhabitants, Tromsø is the largest city in Northern Norway. The study population is considered broadly representative of the Norwegian adult population, with individuals holding a university degree being slightly overrepresented. The analysis presented in this paper is based on a balanced sub-sample drawn from the sixth wave conducted in 2007/08 (n = 12,981, aged 30 and above), and the seventh wave conducted in 2015/16 (n = 21,083, aged 40 and above). The design of the Tromsø Study is described in detail elsewhere [24]. The study was approved by the regional committee for Medical and Health Research Ethics (ID 2016/607). All participants gave written informed consent before admission.

### 2.2 Participants

Out of 5,685 individuals who participated in both waves and were below the upper retirement age in Norway (70 years) at follow-up, we excluded: 1,253 individuals who did not work full-time at baseline; 546 who reported one or more health shocks prior to baseline; 42 who, at baseline, had reported severe problems on at least one of the five health dimensions in the EQ-5D-3L descriptive system [25], and; 4 individuals who reported to be studying or in military service. Based on these criteria, we analysed a sample of 3,840 healthy individuals who were working full time at baseline.

### 2.3 Variables

#### 2.3.1 Outcome

The outcome variable is LMP at follow-up, with three categories: full time, part time and not working. The not-working category included a variety of sub-categories: unemployment, early retirement, disability recipient, work assessment allowance, family income supplement and unpaid domestic work. In our main analysis, we combined the part-time and not-working categories, both of which reflect *reductions* in LMP from full-time work at baseline.

#### 2.3.2 Resilience

An abbreviated version of the RSA was included in wave 7 of the survey (referred to as the follow-up). We chose three items that represented the personal domain of the RSA, which could be satisfactorily summed together in a single index score. A confirmatory one-factor analysis confirmed a good fit [x^2^_df = 1_ = 0.10, P = 0.76; RMSEA = 0 (95% CI 0–0.013)]. Higher scores on these three items indicated a better adaptation response to life stresses. The items asked about: confidence in personal judgements, the ability to thrive/prosper despite difficulties and the use of personal beliefs to overcome difficult times. Items were rated on a Likert scale (1 = ‘disagree completely’ to 5 = ‘agree completely’). The resilience index score represented the average of these three item scores. Data completeness was high, with only 2% (64) missing values. In the case of one missing value, it was replaced by the average of the individual’s two other item scores, that is average imputations.

Since the RSA variable is measured at follow-up, we included *Locus-of-control* and *optimism* with regard to one’s future health (Health optimism) measured at baseline. Both variables were measured on a 7-point scale (1 disagree completely, 7 agree completely). For LOC the item asked about were: ‘I have sufficient influence on when and how my work should be done’. For health optimism the item asked about were: ‘I have a positive view of my future health’.

#### 2.3.3 Health shocks

Participants were asked to report whether they have, or have had, any of the following health conditions: heart attack, angina, stroke, cancer and psychological problems. Due to their limited numbers, we combined the first three conditions into cardiovascular diseases (CVD). We treat health shocks as binary variables in the analysis. Given that we only included subjects that had *not* reported any of these adversities at baseline, all reported health shocks are assumed to have occurred at some point *between* baseline and follow-up.

#### 2.3.4 Health at baseline

In addition to the effect of health shocks occurring *after* baseline, we expect participants’ health *at* baseline to influence LMP at follow-up. Study participants reported their health-related quality of life (HRQoL) by use of the EQ-5D-3L generic descriptive system, which consists of five dimensions (mobility, self-care, usual activities, pain & discomfort and anxiety & depression), each described along three severity levels (no problem, moderate, severe). We distinguish subjects who reported full health (N = 2436), i.e. no problems on all 5 dimensions (EQ-5D profile 11111), from those reporting a moderate health problem (level 2) along at least one dimension (N = 1404). Within this latter group, the majority reported a health profile with moderate pain and discomfort, and no problems on any of the other dimensions (EQ-5D profile 11121) (N = 871).

#### 2.3.5 General covariates

We controlled for age at follow-up, sex and educational attainment level. The age variable was split into three groups: 40–49; 50–61; 62–69 years. We chose these age bands because Norwegians can combine part-time work while receiving partial pension payments after the age of 62. Educational attainment was categorised into four levels in line with the International Standard Classification of Education (ISCED): primary and secondary school (10 years); upper secondary school (3 years); lower college or university degree (< 4 years); higher college, and; university degree (≥ 4 years).

### 2.4 Statistical analysis

We analyzed the data by using binary logistic regression with several specifications. Model 1 specification includes age, sex, education, health at baseline, and presence of health shocks (each entered as indicator variables). Specification 2 adds RSA, specification 3 adds LOC, and specification 4 adds health optimism.

In addition, to test for possible moderations effects, we estimated three models that allowed interactions between the resilience variables and the health shocks. Calculating marginal effects in nonlinear models can be complicated, because a coefficient can be statistically indistinguishable from zero, although the cross-partial derivative is different from zero. We therefore applied the delta method, suggested by Ai and Norton(2003) [13] for exploring interaction terms in nonlinear models.

To further investigate any differences between those working part-time and not-working, our sensitivity analysis consists of a multinomial logistic model that distinguishes these two non-fulltime outcomes. All results are presented as odds ratios (OR).

## 3. Results

Table 1 shows the sample characteristics by LMP at follow-up. Pearson’s chi-square tests indicate unadjusted associations between the explanatory variables and LMP at follow-up. As expected, reductions in LMP is associated with lower education levels, reduced HRQoL at baseline, and health shocks after baseline, i.e. lower human capital.

**Table 1:** Sample characteristics by labour market participation at follow-up, N = 3840.

	Full-time		Part-time		Not-working		P-value from
	(N = 2885)		(N = 243)		(N = 712)		Chi.Sq tests
**Sex**	N	%	N	%	N	%	< 0.001
Men	1550	53.7	78	32.1	354	49.7	
Women	1335	46.3	165	67.9	358	50.3	
**Age**							< 0.001
40–49	804	27.9	28	1.5	31	4.4	
50–61	1677	58.1	84	34.6	103	14.5	
62–69	404	14.0	131	53.9	578	81.2	
**Educational level** ^**a**^							< 0.001
Primary School (10 years)	340	11.8	38	15.6	160	22.6	
Upper Secondary (3 years)	925	32.1	101	41.6	263	37.2	
University < 4 years	679	23.6	49	20.2	146	20.7	
University ≥ 4 years	936	32.5	55	22.6	138	19.5	
**EQ-5D-3L at baseline** ^**b**^							< 0.001
Full health (11111)	1749	64.3	107	46.7	351	52.9	
Moderate health	969	35.7	122	53.3	313	47.1	
**Individuals with health shock after baseline**							< 0.001
No	2609	90.4	187	77.0	545	76.5	
Yes	276	9.6	56	23.0	167	23.5	
**Diagnosis** ^**c**^ **. % by LMP. Ref: No**							
Heart attack	34	1.2	6	2.5	19	2.7	0.007
Angina	15	0.5	0	0.0	8	1.1	0.080
Stroke	13	0.5	8	3.3	26	3.7	< 0.001
Psychological problems	116	4.0	27	11.1	35	4.9	< 0.001
Cancer	119	4.1	19	7.8	91	12.8	< 0.001

^a^10 missing values on education.

^b^229 missing observations on EQ-5D. Moderate health = all EQ-5D-3L profiles with at least one dimension at level 2. Respondents with at least one dimension at level 3 were excluded.

^c^The number of health shock diagnosis (536) are larger than total number of individuals who have experienced health shocks (499): 465 individuals have experienced 1 shock, 33 have experienced 2 shocks, and 1 reported 5 shocks.

S1 Table in S1 File provides the precise wording of the resilience variables as used in the survey, and their mean values by LMP at follow-up. The low p-values support the expected associations between the mean values in the resilience measures and level of LMP. In S2 Table in S1 File, the correlation matrix for the three resilience measures support that they are all representative of a resilience resource.

Table 2 presents the results of the four specifications of the binary logistic models. To ease the comparing between the models they are introduced stepwise from simplest (Model 1) to full model (Model 4). All four model specifications suggest a similar pattern regarding the impacts of sex, age, education, and health. Women are more likely to leave full time work than men. The much higher odds ratios in the oldest age groups (62–69) is attributed to the entitlements to early retirement in the Norwegian social security system. Higher education is strongly associated with a propensity to continue working full-time. Moderate health problems at baseline, or having experienced a health shock after baseline, are associated with reduced LMP at follow-up.

**Table 2:** Binary models.

Reference: Full-time work	Part time and Not-working											
	Model 1			Model 2			Model 3			Model 4		
	OR	95% CI		OR	95% CI		OR	95% CI		OR	95% CI	
Variables		Lower	Upper		Lower	Upper		Lower	Upper		Lower	Upper
Intercept	0.05***	0.03	0.07	0.12***	0.06	0.24	0.15***	0.07	0.32	0.21***	0.09	0.49
Women	2.01***	1.64	2.47	2.04***	1.66	2.52	1.98***	1.61	2.45	2.00***	1.61	2.48
**Age**: reference 40–49												
Age 50–61	1.50**	1.07	2.14	1.54**	1.10	2.21	1.51**	1.07	2.17	1.57**	1.11	2.27
Age 62–69	30.18***	21.76	42.78	30.96***	22.24	44.05	31.05***	22.23	44.33	33.34***	23.7	48.02
**Education**: ref: Primary 10 years												
Upper secondary 3 years	0.89	0.66	1.20	0.87	0.65	1.18	0.89	0.66	1.21	0.87	0.64	1.19
University <4 years	0.62***	0.45	0.85	0.60***	0.43	0.83	0.62***	0.45	0.86	0.62***	0.44	0.86
University ≥4 years	0.36***	0.26	0.50	0.35***	0.25	0.48	0.37***	0.26	0.51	0.36***	0.26	0.51
**Health** at baseline. Ref: Full health; EQ-5D (11111)												
Moderate health	1.58***	1.29	1.93	1.54***	1.25	1.89	1.58***	1.28	1.94	1.48***	1.19	1.84
**Health shocks** after baseline. Ref: no health shock												
CVD	2.99***	1.85	4.85	2.89***	1.78	4.72	2.91***	1.79	4.76	2.83***	1.73	4.67
Psychological prob.	3.30***	2.16	4.98	3.16***	2.06	4.81	3.18***	2.05	4.87	3.16***	2.02	4.87
Cancer	2.15***	1.50	3.08	2.12***	1.48	3.05	2.10***	1.46	3.04	2.07***	1.42	3.00
**Resilience**												
RSA, at follow-up				0.81***	0.70	0.92	0.82***	0.71	0.95	0.85**	0.73	0.98
Locus of control, at baseline							0.94*	0.88	1.01	0.95	0.89	1.02
Health optimism, at baseline										0.90**	0.83	0.99
AIC	2578.0			2549.6			2499.9			2428.6		

Model 2 shows that individuals with higher levels of RSA is more likely to work full-time at follow-up (OR = 0.81, p <0.01), After including RSA, we note a slight reduction in health shocks coefficients. This reduction could, potentially, indicate that RSA moderate health shocks. Model specification 3 shows that higher levels of LOC also makes individuals more likely to work full-time (OR = 0.94, p <0.10). Finally, Model 4 includes *health optimism*, which also shows that higher levels of *health optimism* increases the likelihood of working full-time at follow-up (OR = 0.90, p <0.05). This association is smaller than RSA but larger than LOC and still persists after adjusting for both. These results suggest that the concept of resilience, as measured in different ways, plays a significant role for individuals’ propensity to continue working.

Table 3 provides the three models that includes interactions between each of the resilience measures and the health shocks. Although not statistically significant, all interactions in Model 2-RSA point in the same consistent direction, i.e. a higher propensity to continue working full-time, particularly in the case of CVD (OR = 0.75) and cancer shocks (OR = 0.78). As for the other two resilience measures, interaction results are mixed.

**Table 3:** Binary models including interactions. Reference: Full-time working.

	Model 2-RSA			Model 3-LOC			Model 4-Hopt		
	Odds ratio	95% CI		Odds ratio	95% CI		Odds ratio	95% CI	
Intercept	0.10 ***	0.05	0.22	0.06 ***	0.04	0.11	0.10 ***	0.05	0.19
Women	2.05 ***	1.66	2.52	1.94 ***	1.57	2.39	2.02 ***	1.63	2.50
**Age:** reference 40–49 years									
Age 50–61	1.54 **	1.10	2.20	1.47 **	1.04	2.10	1.53 **	1.09	2.20
Age 62–69	30.95 ***	22.21	44.08	30.47 ***	21.88	43.39	32.35 ***	23.12	46.30
**Education:** ref. Primary 10 years									
Upper secondary 3 years	0.87	0.64	1.18	0.90	0.67	1.22	0.86	0.63	1.17
University <4 years	0.60 ***	0.43	0.83	0.63 ***	0.45	0.88	0.60 ***	0.43	0.83
University ≥4 years	0.35 ***	0.25	0.48	0.38 ***	0.27	0.52	0.35 ***	0.25	0.49
**Health** at baseline. Ref: Full health; EQ-5D (11111)									
Moderate health	1.53 ***	1.25	1.88	1.60 ***	1.30	1.97	1.46 ***	1.18	1.82
**Health shocks** after baseline. Ref: no health shock									
CVD	9.96	0.42	244.0	3.06	0.41	23.07	11.67 **	1.21	135.09
Psychological prob.	4.16	0.45	35.28	2.76	0.49	14.23	2.06	0.28	13.83
Cancer	5.89	0.64	62.35	4.02 **	1.03	16.43	2.08	0.42	10.32
**Resilience**									
RSA, at follow-up	0.83 **	0.72	0.97						
Locus of control at baseline				0.94 *	0.88	1.01			
Health optimism at baseline							0.88 ***	0.80	0.97
**Interactions**									
CVD*RSA	0.75	0.36	1.56						
Psych.Prob*RSA	0.93	0.54	1.63						
Cancer*RSA	0.78	0.45	1.32						
CVD*Locus of control				1.00	0.70	1.42			
Psych.Prob*Locus of control				1.04	0.77	1.42			
Cancer*Locus of control				0.89	0.69	1.13			
CVD*Health optimism							0.77	0.49	1.17
Psych.Prob*Health optimism							1.10	0.76	1.61
Cancer*Health optimism							1.00	0.74	1.34
AIC	2554.1			2527.3			2470.3		

### 3.1 Sensitivity analysis

Our results demonstrate that RSA (at follow-up) and health optimism (at baseline) is positively associated with a propensity to work full-time at follow-up. To further investigate these effects we split those who are not working full-time into part-time (n = 243), and not-working (n = 712). The S3-S5 Tables in S1 File presents the multinomial logit models, which has the same specifications as Model 2, 3 and 4.

We observe a similar pattern across these three multinomial models for sex, age, education, health at baseline and health shocks. S3 Table in S1 File Model 2 contains the first specification where only RSA is included of the resilience measures. Note the much stronger RSA association in the not-working group (OR = 0.76, p <0.01), as compared with the part-time group (OR = 0.91). S4 Table in S1 File Model 3 further includes LOC. Again, the direction of the associations are similar to the binary model, but only significant for the not-working category: RSA (OR = 0.78, p<0.05) and LOC (OR = 0.93, p<0.10). Finally, S5 Table in S1 File Model 4 includes health optimism. This indicator is only statistically significant in the not-working category (OR = 0.89, p <0.05). Thus, the multinomial models indicate that lower resilience contributes to explain why individuals opt not to work at all, but *not* why individuals reduce their LMP from full-time to part-time.

## 4. Discussion

The purpose of this longitudinal study was to investigate the hypotheses that resilience helps individuals in sustaining their level of labour market participation (LMP) and if resilience operates as a protective factor against a health shock. We used a validated resilience measure as well as two related measures; locus of control, and health optimism, to investigate these hypotheses.

We find that more personal resilience resources are positively associated with maintaining full-time work. The results were consistent after controlling for sex, age, educational attainment and health. In other words, this indicate that higher level of resilience helps individuals in sustaining their level of LMP, independent of their human capital. The results converge with the PsyCap theory, which does not require any adversity for resilience to be meaningful. This finding lends support to earlier studies, that suggest resilience is positively associated with work engagement, [14] job performance [15] and job satisfaction [16]. Although these earlier studies do not directly confirm each other, they point in the same direction in terms of job sustainability.

Psychosomatic studies have shown that higher resilience may counteract ischemic pain and stressful experiences [9], as well as hopelessness and depressive symptoms [17]. However, our results did not support the hypothesis that higher resilience score operates as a protective factor against health shocks. As such, our result deviate from Schurer’s study [12] which used LOC as a proxy for resilience, showing that non-resilient individuals are more likely to reduce their labour supply after experiencing a health shock. The following reasons may explain the deviating results. First, while economists do not emphasise the difference between LOC and resilience, psychologists argue that these two concepts are different and subsequently claim that studies measure different concepts. Second, the previous study was conducted in different institutional setting. Norway has an extensive social insurance system including generous sickness benefit schemes. Such financial protection affords people not to work full time after experiencing a health shock, i.e. they do not have the same financial incentive to utilize their psychological capital. In other words, the more an attractive universal financial protection scheme make people decide *not* to work when their health deteriorates, the less important becomes individual variations in their resilience for explaining why people keep on working despite experiencing a health shock.

Our study have several strengths. First, it is a longitudinal study with an eight year interval. Second, we use comprehensive measures of respondents’ health; at baseline measured by the most widely applied generic preference based descriptive system for health-related quality of life (EQ-5D-3L), and after baseline; self-reported experiences of three sets of health shocks (cardiovascular, cancer, mental health). Third, we adjust for socio-economic differences measured by four levels of educational attainment.

A potential weakness is that our key measure of resilience (RSA) was collected at follow-up, while health shocks occurred between the baseline and the follow-up. Thus, survey participants’ resilience levels might have been affected by experiencing a health shock. However, as some individuals might strengthen rather than weakening their resilience resources after an adversity, this need not be a major limitation. Also, the RSA seems to capture personal resources of the individual that are of a highly stable character, and it also correlates strongly with stable Big Five personality traits, in particular neuroticism [18]. In a Norwegian general population study the four month test-retest stability correlation of the RSA dimension used in the current study was very high (*r* = .79) [19]. Still, acknowledging bias in measuring resilience at follow-up, we included two additional indicators measured at baseline as representatives of resilience. The correlation tests between our resilience measures show that these measures points in the same direction and provide support to the hypothesis that having more rather than less resilience resources heightens the likelihood to keep on working.

## 5. Conclusions

Higher levels of resilience is associated with full-time work as individuals age. However, our results did not provide evidence to support the hypothesis that resilience moderates the effect of health shocks on LMP. This might be explained by an institutional context whereby people are fortunate to rely on universal sickness benefit schemes rather than having to activate a key attribute of their individual psychological capital.

## Supporting information

S1 File (DOCX)
