# Supplementary material for: Who keeps on working? The importance of resilience for labour market participation
Source: PLoS One. 2021 Oct 13;16(10):e0258444. doi: 10.1371/journal.pone.0258444 (PMC8513899; doi:10.1371/journal.pone.0258444)
Supplement: S1 File — (DOCX) [file pone.0258444.s001.docx]

# A. Appendix

Supporting tables for the manuscript.

## A.1 Tables

| S1 Table: Resilience measures, as described in the questionnaires. Mean scores (and SD) by job-participation category. | | | | |
| --- | --- | --- | --- | --- |
| **Resilience Scale for Adults (RSA)** |  |  |  |  |
| Please indicate how well the followings statements describe you. (1 disagree completely, 5 agree completely) | Full-Time (N=2885) | Part-Time (N=243) | Not-Working (N=712) | P-value from Anova |
| Confidence in own judgements/decisions. | 4.31 (0.76) | 4.26 (0.79) | 4.22 (0.80) |  |
| Aptitude to thrive/prosper despite adversity | 4.26 (0.82) | 4.24 (0.83) | 4.15 (0.85) |  |
| Able to overcome difficulties due to positive self-beliefs | 4.21 (0.81) | 4.15 (0.80) | 4.13 (0.83) |  |
| Average RSA | 4.26 (0.71) | 4.22 (0.73) | 4.17 (0.74) | 0.007 |
| Statements **at baseline** were rated on a 7-point scale  (1 disagree completely, 7 agree completely) |  |  |  |  |
|  |  |  |  |  |
| **Locus of control (LOC)**  I have sufficient influence on when and how my work should be done. | 5.48 (1.47) | 5.34 (1.59) | 5.31 (1.56) | 0.019 |
| **Health optimism (Hopt)** |  |  |  |  |
| I have a positive view of my future health. | 5.51 (1.18) | 5.30 (1.29) | 5.27 (1.31) | <0.001 |

| S2 Table: Correlation matrix for the resilience measures. | | | |
| --- | --- | --- | --- |
| Variables | RSA | LOC | Hopt |
| RSA, at follow-up | 1 |  |  |
| LOC, at base line | .10*** | 1 |  |
| Hopt, at base line | .20*** | .18*** | 1 |

| S3 Table: **Model 2.** | | | | | | | | | | |
| --- | --- | --- | --- | --- | --- | --- | --- | --- | --- | --- |
| Reference: Full-time working |  | Part-time | | | |  | Not-working | | | |
|  |  | Betas | Lower 95% CI | Odds ratio | Upper 95% CI |  | Betas | Lower 95% CI | Odds ratio | Upper 95% CI |
| Intercept |  | -3.87 | 0.01 | 0.02*** | 0.06 |  | -2.34 | 0.04 | 0.10*** | 0.22 |
| Women |  | 1.11 | 2.22 | 3.05*** | 4.19 |  | 0.54 | 1.36 | 1.72*** | 2.16 |
| **Age**: reference 40-49 |  |  |  |  |  |  |  |  |  |  |
| Age 50-61 |  | 0.39 | 0.91 | 1.47 | 2.39 |  | 0.49 | 1.01 | 1.62** | 2.61 |
| Age 62-69 |  | 2.55 | 7.86 | 12.74*** | 20.67 |  | 3.85 | 30.18 | 47.22*** | 73.87 |
| **Education**: ref: Primary 10 years |  |  |  |  |  |  |  |  |  |  |
| Upper secondary 3 years |  | 0.12 | 0.72 | 1.13 | 1.78 |  | -0.26 | 0.56 | 0.77 | 1.08 |
| University <4 years |  | -0.36 | 0.42 | 0.69 | 1.15 |  | -0.59 | 0.39 | 0.55*** | 0.79 |
| University ≥4 years |  | -0.73 | 0.29 | 0.48*** | 0.79 |  | -1.20 | 0.21 | 0.30*** | 0.43 |
| **Health** at baseline. Ref: Full health; EQ-5D (11111) |  |  |  |  |  |  |  |  |  |  |
| Moderate health |  | 0.51 | 1.23 | 1.66*** | 2.25 |  | 0.40 | 1.18 | 1.49*** | 1.87 |
| **Health shocks** after baseline. Ref: no health shock |  |  |  |  |  |  |  |  |  |  |
| CVD |  | 1.04 | 1.43 | 2.83*** | 5.61 |  | 1.08 | 1.74 | 2.93*** | 4.95 |
| Psychological prob. |  | 1.42 | 2.49 | 4.15*** | 6.92 |  | 0.89 | 1.43 | 2.43*** | 4.13 |
| Cancer |  | 0.36 | 0.81 | 1.43 | 2.55 |  | 0.89 | 1.65 | 2.44*** | 3.60 |
| **Resilience** |  |  |  |  |  |  |  |  |  |  |
| RSA at follow up |  | -0.10 | 0.74 | 0.91 | 1.12 |  | -0.27 | 0.65 | 0.76*** | 0.89 |
| Note: *p<0.1, **p<0.05, ***p<0.01 | | | | | | | | | | |

| S4 Table. **Model 3.** | | | | | | | | | | |
| --- | --- | --- | --- | --- | --- | --- | --- | --- | --- | --- |
| Reference: Full-time working |  | Part-time | | | |  | Not-working | | | |
|  |  | Betas | Lower Upper 95% CI | Odds ratio | Upper 95% CI |  | Betas | Lower 95% CI | Odds ratio | Upper 95% CI. |
| Intercept |  | -3.76 | 0.01 | 0.02*** | 0.08 |  | -2.09 | 0.05 | 0.12*** | 0.31 |
| Women |  | 1.11 | 2.21 | 3.05*** | 4.21 |  | 0.50 | 1.30 | 1.64*** | 2.08 |
| **Age**: reference 40-49 |  |  |  |  |  |  |  |  |  |  |
| Age 50-61 |  | 0.41 | 0.92 | 1.51* | 2.47 |  | 0.42 | 0.95 | 1.53* | 2.46 |
| Age 62-69 |  | 2.57 | 7.97 | 13.04*** | 21.33 |  | 3.84 | 29.69 | 46.49*** | 72.80 |
| **Education**: ref: Primary 10 years |  |  |  |  |  |  |  |  |  |  |
| Upper secondary 3 years |  | 0.12 | 0.71 | 1.12 | 1.77 |  | -0.23 | 0.57 | 0.80 | 1.12 |
| University <4 years |  | -0.39 | 0.40 | 0.68 | 1.13 |  | -0.53 | 0.41 | 0.59*** | 0.85 |
| University ≥4 years |  | -0.72 | 0.30 | 0.49*** | 0.80 |  | -1.13 | 0.22 | 0.32*** | 0.47 |
| **Health** at baseline. Ref: Full health; EQ-5D (11111) |  |  |  |  |  |  |  |  |  |  |
| Moderate health |  | 0.52 | 1.24 | 1.68*** | 2.29 |  | 0.42 | 1.21 | 1.53*** | 1.93 |
| **Health shocks** after baseline. Ref: no health shock |  |  |  |  |  |  |  |  |  |  |
| CVD |  | 1.07 | 1.47 | 2.91*** | 5.76 |  | 1.07 | 1.72 | 2.92*** | 4.95 |
| Psychological prob. |  | 1.38 | 2.35 | 3.99*** | 6.77 |  | 0.94 | 1.49 | 2.55*** | 4.38 |
| Cancer |  | 0.24 | 0.70 | 1.28 | 2.34 |  | 0.91 | 1.68 | 2.49*** | 3.70 |
| **Resilience** |  |  |  |  |  |  |  |  |  |  |
| RSA at follow up |  | -0.08 | 0.75 | 0.93 | 1.15 |  | -0.25 | 0.66 | 0.78*** | 0.91 |
| LOC at baseline |  | -0.04 | 0.87 | 0.96 | 1.06 |  | -0.07 | 0.87 | 0.93* | 1.01 |
| Note: *p<0.1, **p<0.05, ***p<0.01 | | | | | | | | | | |

| S5 Table. **Model 4.** | | | | | | | | | | |  |
| --- | --- | --- | --- | --- | --- | --- | --- | --- | --- | --- | --- |
| Reference: Full-time working |  | Part-time | | | |  | Not-working | | | | |
|  |  | Betas | Lower 95% CI. | Odds ratio | Upper 95% CI |  | Betas | Lower 95% CI | Odds ratio | Upper 95% CI. | |
| Intercept |  | -3.45 | 0.01 | 0.03*** | 0.12 |  | -1.71 | 0.07 | 0.18*** | 0.48 | |
| Women |  | 1.12 | 2.20 | 3.06*** | 4.27 |  | 0.51 | 1.31 | 1.66*** | 2.11 | |
| **Age**: reference 40-49 |  |  |  |  |  |  |  |  |  |  | |
| Age 50-61 |  | 0.41 | 0.91 | 1.51 | 2.50 |  | 0.49 | 1.00 | 1.63** | 2.65 | |
| Age 62-69 |  | 2.62 | 8.28 | 13.70*** | 22.65 |  | 3.92 | 31.90 | 50.48*** | 79.90 | |
| **Education**: ref: Primary 10 years |  |  |  |  |  |  |  |  |  |  | |
| Upper secondary 3 years |  | 0.08 | 0.67 | 1.08 | 1.72 |  | -0.24 | 0.56 | 0.79 | 1.10 | |
| University <4 years |  | -0.34 | 0.42 | 0.71 | 1.20 |  | -0.56 | 0.39 | 0.57*** | 0.83 | |
| University ≥4 years |  | -0.67 | 0.31 | 0.51*** | 0.85 |  | -1.18 | 0.21 | 0.31*** | 0.45 | |
| **Health** at baseline. Ref: Full health; EQ-5D (11111) |  |  |  |  |  |  |  |  |  |  | |
| Moderate health |  | 0.47 | 1.16 | 1.60*** | 2.22 |  | 0.35 | 1.11 | 1.43*** | 1.82 | |
| **Health shocks** after baseline. Ref: no health shock |  |  |  |  |  |  |  |  |  |  | |
| CVD |  | 1.00 | 1.34 | 2.72*** | 5.51 |  | 1.06 | 1.69 | 2.89*** | 4.93 | |
| Psychological prob. |  | 1.38 | 2.31 | 3.96*** | 6.78 |  | 0.94 | 1.48 | 2.55*** | 4.40 | |
| Cancer |  | 0.20 | 0.66 | 1.22 | 2.29 |  | 0.90 | 1.65 | 2.46*** | 3.68 | |
| **Resilience** |  |  |  |  |  |  |  |  |  |  | |
| RSA, at follow-up |  | -0.05 | 0.76 | 0.95 | 1.18 |  | -0.22 | 0.68 | 0.80*** | 0.94 | |
| LOC, at baseline |  | -0.05 | 0.86 | 0.95 | 1.05 |  | -0.05 | 0.89 | 0.96 | 1.03 | |
| Hopt, at baseline |  | -0.07 | 0.81 | 0.93 | 1.06 |  | -0.12 | 0.80 | 0.89** | 0.98 | |
| Note: *p<0.1, **p<0.05, ***p<0.01 | | | | | | | | | | |  |
